# Global Research Trends in Shock Wave for Therapy from 1990 to 2019: A Bibliometric and Visualized Study

**DOI:** 10.1155/2021/3802319

**Published:** 2021-01-07

**Authors:** Qingxi Zhang, Yu Zhao, Dan Xing, Jianhao Lin

**Affiliations:** ^1^Arthritis Clinic & Research Center, Peking University People's Hospital, Peking University, Beijing, China; ^2^Arthritis Institute, Peking University, Beijing, China

## Abstract

**Objective:**

The publications of application and development of shock wave therapy showed consistent growth. The aim of this study was to investigate the global status and trends in the shock wave therapy field.

**Methods:**

Publications about shock wave therapy from 1990 to 2019 were collected from the Web of Science database. The data were studied and indexed by using bibliometric methodology. For a visualized study, VOSviewer software was used to conduct bibliographic coupling analysis, coauthorship analysis, cocitation analysis, and co-occurrence analysis and to analyze the publication trends in shock wave therapy.

**Results:**

A total of 3,274 articles were included. The number of publications was increasing per year globally. The USA made the largest contributions to the global research with the most citations (the highest *h*-index). The Journal of Urology had the highest publication number. The University of California System was the most contributive institution. Studies could be divided into seven clusters: urology, hepatology, cardiology, orthopedics, mechanism research of shock wave therapy, andrology, and principle of shock wave therapy. Orthopedics, andrology, and mechanism research of shock wave therapy could be the next hot topics in this field.

**Conclusions:**

Base on the trends, shock wave therapy is the theme of a globally active research field which keeps developing and extends from bench to bedside.

## 1. Introduction

Extracorporeal shock waves have been introduced to medical therapy approximately 40 years since its first treatment for kidney stones as a noninvasive method (lithotripsy) [1]. Shock waves are sonic pulses, characterized by an initial increase, reaching a positive peak of up to 100 MPa within 10 ns, followed by a negative amplitude of up to -10 MPa and a total life cycle of less than 10 s [2]. Four common different types of shock wave generators are used in the medical field today, and they differ from one another primarily in the way they generate the shock waves. They are the electrohydraulic generator, electromagnetic generator, ballistic generator, and piezoelectric generator [3].

Initially, shock waves were focused on disintegrating different kinds of stones (urinary tract, biliary, and salivary). Then, many studies were undertaken to assess the effects of shock waves on similar hard tissues such as bone, contiguous, and near bone tissues subsequently [4, 5]. The first shock wave treatment of fracture nonunions and delayed unions in humans was successfully presented in 1991, for the reason that shock waves can promote osteogenesis, especially the elaboration of callus [5]. At the same time, with the advantages of shock wave therapy, avoidance of surgeries, safety, and reasonable cost, it has been widely used in many other musculoskeletal disorders, such as tendinitis calcarea and epicondylitis [6]. In 2000, Wang et al. found that shock waves enhance neovascularization on the tendon-bone junction, and this enlightening result expands the future development in this field [7]. Up to now, shock waves have been applied in clinics for several decades and have demonstrated beneficial effects on urology, orthopedics (nonunion fractures and ischemia-induced tissue necrosis), dermatology (calcinosis cutis, ulcers, and burn wounds), neurology (unresponsive wakefulness syndrome), and cardiology (ischemic hearts and limbs) [8–16]. However, some of these results remain to be verified by high-quality studies of the clinical results. Furthermore, in contrast to the relatively well-documented effects of shock waves, very little was known regarding the underlying mechanisms at the cellular and molecular level. Therefore, some researchers paid close attention to the basic study. For example, the mechanical stimulus of the shock wave caused the release of angiogenic exosomes which could develop an innovative approach for the regeneration of the ischemic myocardium [16]. Chen et al. reported that the bone marrow mesenchymal stem cells treated by an extracorporeal shock wave can enhance bone defect healing [17]. These results of basic research partially verified the clinical results and pointed out the direction for future study. But it is worth noting that the global development trends of shock waves have not been well studied yet. Thus, it is necessary to summarize the research status of shock wave therapy and predict significative keywords and trends.

Publication is an important indicator to evaluate the quality of scientific research in a certain field. Through bibliometric analysis, which is based on the literature databases and literature metrology characteristics to qualitatively and quantitatively evaluate trends in research activity over time, it is helpful to predict the development of a certain field and compare the contributions of scholars, institutions, countries, and journals [18]. It is also valuable to develop clinical policy and make guidelines [19]. Besides, this practicable analysis has been effectively used in diverse areas, which makes the study field unambiguous [20–22]. Nonetheless, to our knowledge, the quantity and quality of shock wave therapy research production have not been reported. Therefore, the purpose of the present study was to evaluate the global status and trends of shock wave therapy.

Through bibliometric analysis, we uncovered the research trend of the shock wave therapy field and further predicted its possible hotspots in the future. The researchers and relevant departments commendably understood the current research status in the field.

## 2. Materials and Methodology

### 2.1. Data Source

The data source of this study is the publication information from the Web of Science (WoS) Core Collection which was deemed as the optimal database for bibliometrics [23].

### 2.2. Search Strategy

All the information of the publications was taken from the Web of Science, with a database expiration of 31 December 2019. In this study, the search terms were as follows: theme = shock wave∗ AND therapy AND publishing year = (1980–2019) AND Language = (English) AND Document types = (ARTICLE OR REVIEW). By collecting the region/country data on the Web of Science, we refined the search for certain countries or regions.

### 2.3. Data Collection

The entire records of these publications, including the year of publication, authors' names, title, name of publishing journal, affiliations, nationalities, keywords, and abstract, were saved as TXT files from the WoS database and then read by Microsoft Excel 2017. Two of the authors separately checked and extracted the data of these publications. Any disagreement was resolved by discussion or turning to help from experts to reach a consensus. Finally, the two authors analyzed the data in Microsoft Excel 2017.

### 2.4. Bibliometric Analysis

The intrinsic function of WoS was used to describe the before-mentioned basic features of eligible publications. The *h*-index was used to assess the impact of scientific research. The index of *h* implied that a scholar or country has published *h* papers each of which has been cited in other publications at least *h* times. Thus, the *h*-index reflects both the number of publications and the number of citations per publication [24].

### 2.5. Visualized Analysis

VOSviewer (Leiden University, Leiden, The Netherlands), as a software tool, was used for constructing and visualizing bibliometric networks of the publications [25]. We used the VOSviewer for bibliographic coupling, coauthorship, cocitation, and co-occurrence analyses. The following options were selected during the import: “create a map based on bibliographic data,” “read data from bibliographic database files,” “type of analysis: bibliographic coupling,” “unit of analysis: sources, organizations, countries,” and “counting method: full counting”; “create a map based on bibliographic data,” “read data from bibliographic database files,” “type of analysis: co-authorship,” “unit of analysis: authors, organizations, countries,” and “counting method: full counting”; “create a map based on bibliographic data,” “read data from bibliographic database files,” “type of analysis: co-citation,” “unit of analysis: cited references, cited sources,” and “counting method: full counting”; and “create a map based on bibliographic data,” “read data from bibliographic database files,” “type of analysis: co-occurrence,” “unit of analysis: all keywords,” “counting method: full counting,” “network visualization,” and “overlay visualization”. Using these settings, the software analyzes and visualizes them in the form of bubble maps.

## 3. Results

### 3.1. Trends in Global Publication

#### 3.1.1. Amount of Global Publication

**Figure 1:**
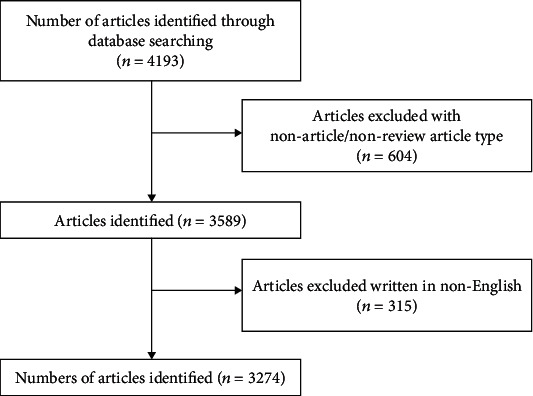
Flowchart of the article selection process used in the study.

**Figure 2:**
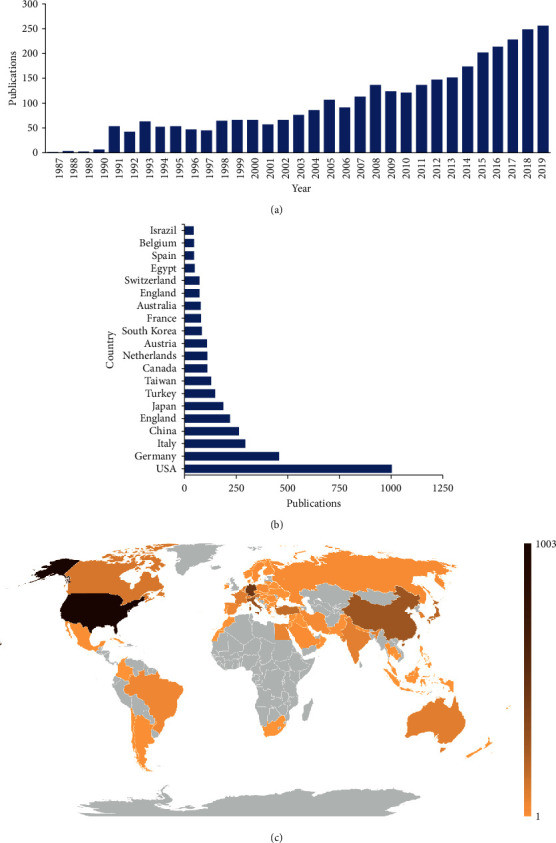
Global trends and countries contributing to shock wave therapy. (a) The single-year publication numbers in the past 33 years related to shock wave therapy. (b) The sum of shock wave therapy research-related articles from the top 20 countries. (c) World map showing the distribution of shock wave therapy research.

The search outcome is shown in the flowchart (Figure 1). There were a total of 3,274 articles that met the search criteria from 1987 to 2019. Globally, the number of publications about shock wave therapy showed a positive growth trend, from 1 in 1987 to 255 in 2019 (Figure 2(a)). This indicates that shock wave therapy research is an exciting and rapidly developing field.

#### 3.1.2. Contribution of Countries

A total of 79 countries and regions made contributions to the world's shock wave therapy publications. Among these countries, the USA published the largest number of articles (1,003, 30.64%), followed by Germany (458, 13.99%), Italy (294, 8.98%), China (264, 8.06%), and England (220, 6.72%) (Figures 2(b) and 2(c)).

### 3.2. Quality of Publications of Different Countries

#### 3.2.1. Total Citation Frequency

**Figure 3:**
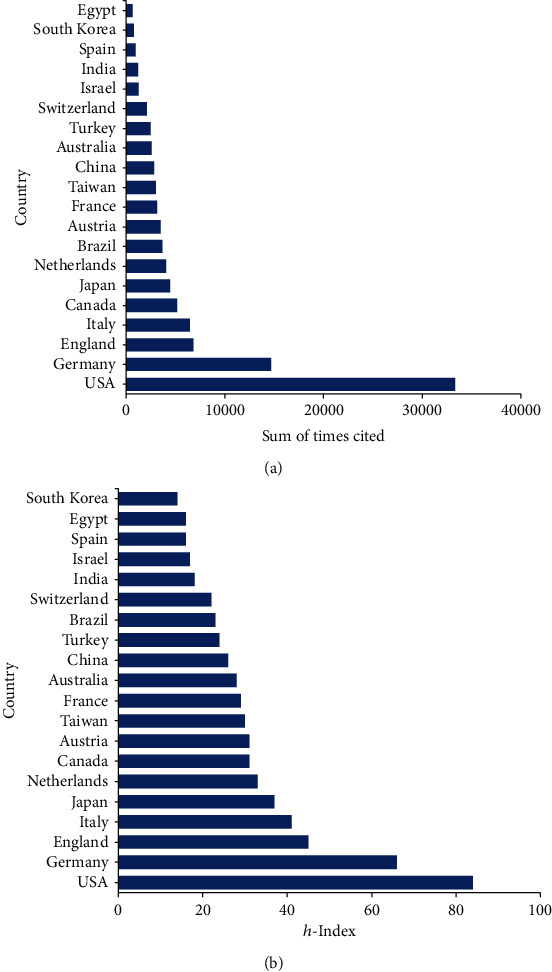
Citation frequency and *h*-index levels of different countries. (a) The total citations for shock wave therapy articles from different countries. (b) The *h*-index of publications in the different countries.

Publications from the USA had the highest total citation frequencies (33,326). Then, Germany ranked second in total citation frequencies (14,679), followed by England (6,814), Italy (6,491), and Canada (5,167) (Figure 3(a)).

#### 3.2.2. *h*-Index

The involved articles from the USA had the highest *h*-index (84), followed by Germany (66), England (45), Italy (41), and Japan (37) (Figure 3(b)).

### 3.3. Analysis of Global Publications

#### 3.3.1. Journal Analysis

**Figure 4:**
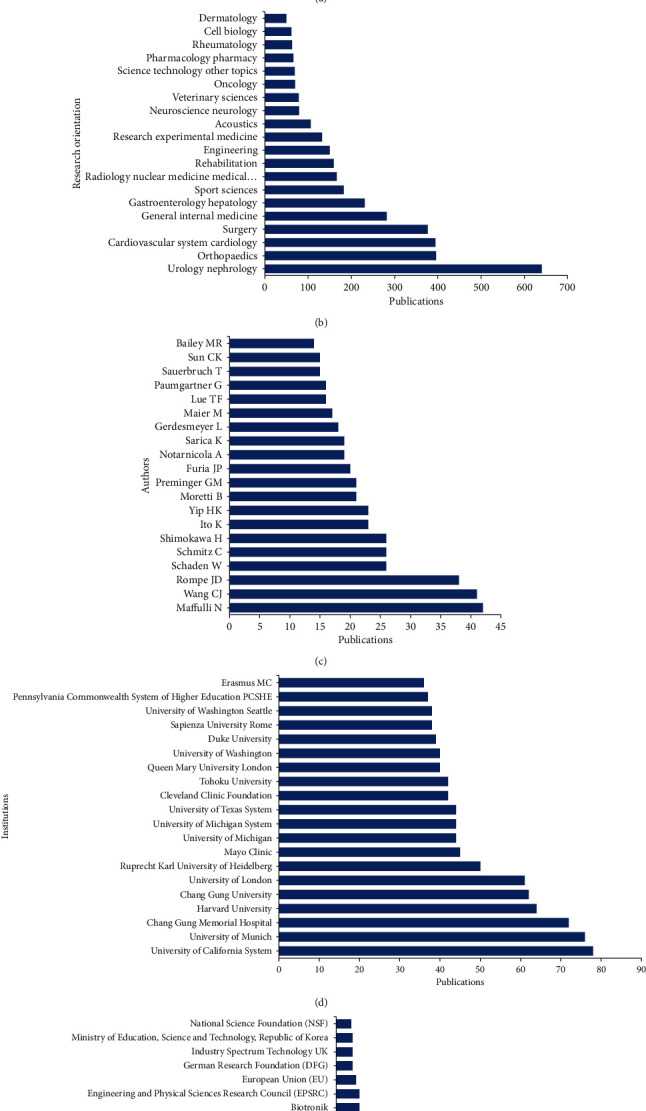
High-contribution journals, research orientations, high-impact institutions, authors, and funds of global research about shock wave therapy: (a) the top-20 research journals in the world; (b) the sum of research orientations in the world; (c) the high-impact authors in the world; (d) the high-impact institutions in the world; (e) the major contribution funds in the world.

The Journal of Urology (impact factor (IF) = 5.647, 2018) published the most studies with 132 publications. There are 69 articles in Ultrasound in Medicine and Biology (IF = 2.205, 2018), 62 articles in Urology (IF = 1.861, 2018), 58 articles in the Journal of Endourology (IF = 2.267, 2018), and 50 articles in Pacing and Clinical Electrophysiology (PACE) on shock wave therapy (IF = 1.343, 2018). The top 20 journals that published the most articles are listed in Figure 4(a).

#### 3.3.2. Research Orientations

As shown in Figure 4(b), urology and nephrology are the most popular research fields (640, 19.55%) and orthopedics ranked second (396, 12.10%), followed by cardiovascular system cardiology (394, 12.03%), surgery (377, 11.52%), and general internal medicine (282, 8.61%).

#### 3.3.3. Authors

Figure 4(c) shows the distribution of authors related to shock wave therapy. Maffylli published the most research on this field with 42 papers, followed by Wang with 41 papers and Rompe with 38 papers.

#### 3.3.4. Institutional Output

Figure 4(d) shows the contributive institution distribution of publications related to shock wave therapy. The University of California System published the most papers (78 papers), then the University of Munich (76 papers), while Chang Gung Memorial Hospital ranked third (72 papers).

#### 3.3.5. Funding Source

The top 20 funding bodies are shown in Figure 4(e). The United States Department of Health and Human Services providing financial support for 153 papers ranked the first, followed by the National Institutes of Health (NIH) USA (150 papers) and the National Nature Science Foundation of China (92 papers).

### 3.4. Bibliographic Coupling Analysis

#### 3.4.1. Journals

**Figure 5:**
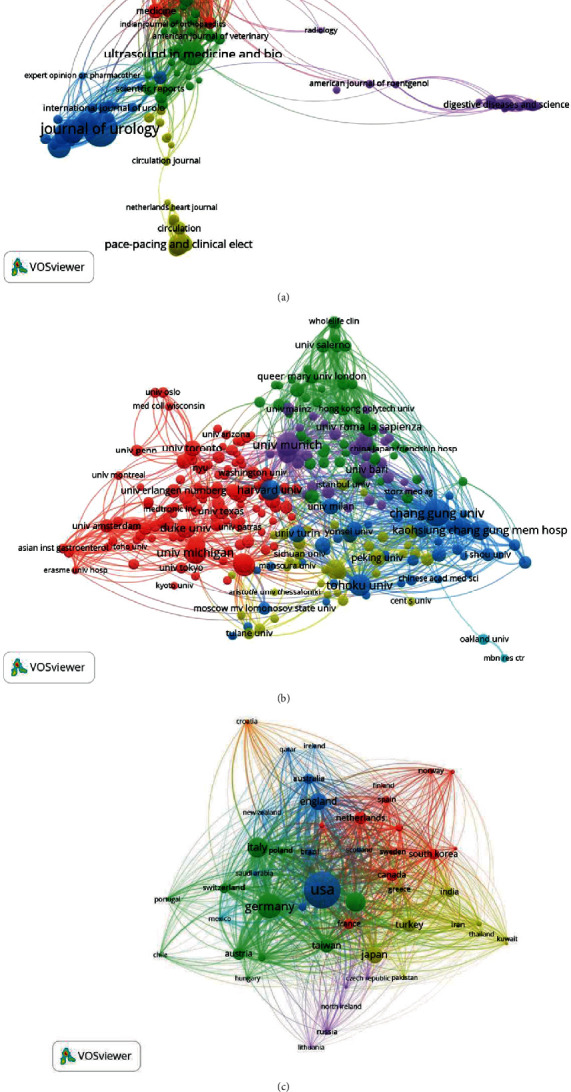
Bibliographic coupling analysis of global research about shock wave therapy. (a) Mapping of the 142 identified journals on shock wave therapy. (b) Mapping of the 292 institutions on shock wave therapy. (c) Mapping of the 50 countries on shock wave therapy. The line between two points in the figure represents that two journals/institutions/countries had established a similarity relationship. The thicker the line, the closer the link between the two journals/institutions/countries.

Bibliographic coupling is a well-established measure that uses citation analysis to establish a similarity relationship between documents. We use the VOSviewer to analyze the journal names in total publications.  Figure 5(a) shows that 142 journals appeared in total link strength (TLS). The top five journals with large total link strengths were as follows: Ultrasound in Medicine and Biology (TLS = 18,337), American Journal of Sports Medicine (TLS = 14,964), Journal of Urology (TLS = 13,144), Clinical Orthopaedics and Related Research (TLS = 12,912), and Foot & Ankle International (TLS = 11,265).

#### 3.4.2. Institutions

All the papers were reported in 293 institutions and were included and analyzed via VOSviewer (the minimum number of publications of an institution was over five). The top five institutions with large total link strength were the following: Chang Gung University (TLS = 49,349), University of Munich (TLS = 35,092), Tohoku University (TLS = 27,436), University Roma La Sapienza (TLS = 25,905), and Kaohsiung Chang Gung Memorial Hospital (TLS = 24,972) (Figure 5(b)).

#### 3.4.3. Countries

Papers identified in the 50 countries were analyzed using VOSviewer (the minimum number of publications from a country was over five). The top five countries with large total link strength were the following: USA (TLS = 334,782), Germany (TLS = 215,849), Italy (TLS = 184,991), England (TLS = 130,235), and China (TLS = 129,638) (Figure 5(c)).

### 3.5. Coauthorship Analysis

#### 3.5.1. Authors

**Figure 6:**
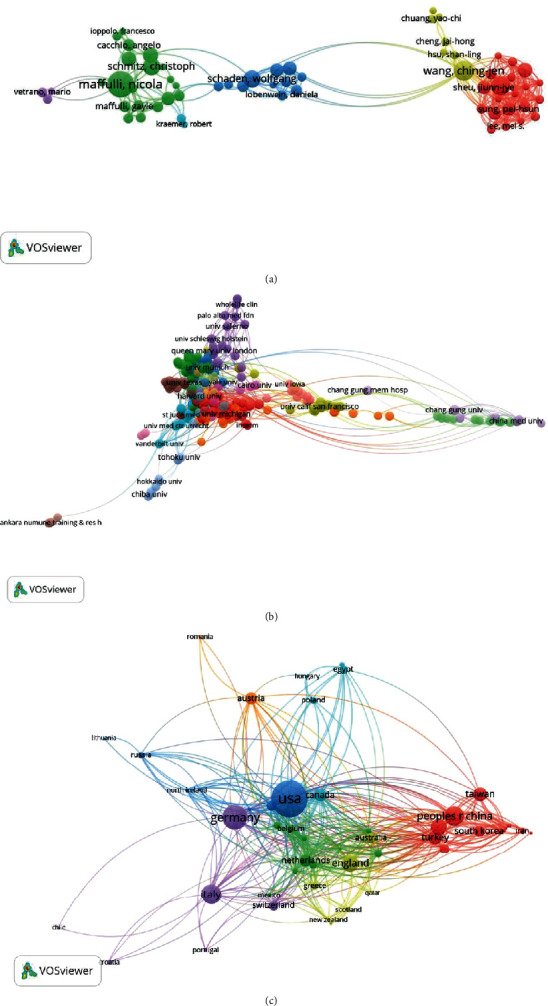
Coauthorship analysis of research about shock wave therapy. (a) Mapping of the coauthorship analysis of 62 authors on shock wave therapy. (b) Mapping of the coauthorship analysis of 273 institutions on shock wave therapy. (c) Mapping of the coauthorship analysis of 50 countries on shock wave therapy. The size of the points represents the coauthorship frequency. The line between two points in the figure represents that two authors/institutions/countries had established collaboration. The thicker the line, the closer the collaboration between the two authors/institutions/countries.

Coauthor analysis refers to the establishment of the relationship between items according to the number of coauthors [26, 27]. A total of 62 authors were identified and analyzed through the VOSviewer (the minimum number of documents from an author was over five). The top five authors with large total link strengths were as follows: Yip, Hon-kan (TLS = 159); Sun, Cheuk-Kwan (TLS = 104); Sung, Pei-Hsun (TLS = 103); Sheu, Jiunn-Jye (TLS = 96); and Maffulli, Nicola (TLS = 93) (Figure 6(a)).

#### 3.5.2. Institutions

Studies identified in the 273 institutions were analyzed using the VOSviewer (the minimum number of publications from an institution was over five). The top five institutions with large total link strength were the following: Chang Gung University (TLS = 123), Kaohsiung Chang Gung Memorial Hospital (TLS = 93), China Medical University (TLS = 70), University of Salerno (TLS = 63), and University of Pittsburgh (TLS = 57) (Figure 6(b)).

#### 3.5.3. Countries

Publications (the minimum number of studies from a country was over five) identified in the 50 countries were analyzed using the VOSviewer (Figure 6(c)). The top five countries with large total link strength were the following: USA (TLS = 447), Germany (TLS = 323), England (TLS = 206), Italy (TLS = 285), and Netherlands (TLS = 104).

### 3.6. Cocitation Analysis

#### 3.6.1. Publications

**Figure 7:**
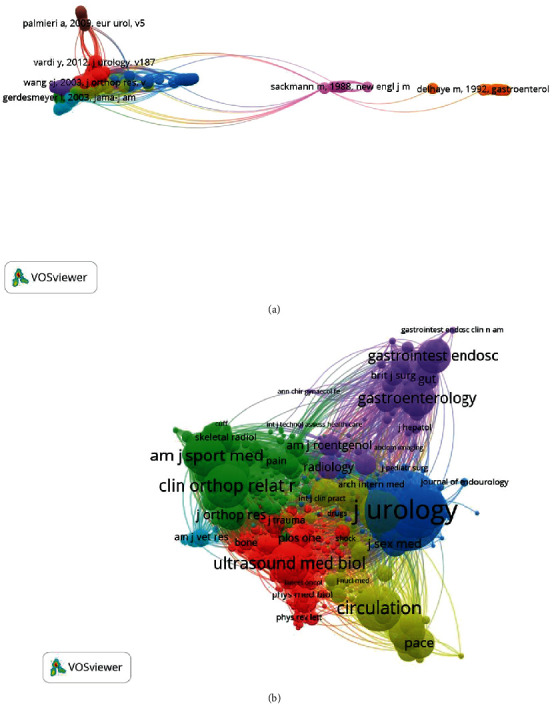
Mapping of cocitation related to shock wave therapy. (a) Mapping of cocited references related to the field. (The 498 points with different colors represent the 435 cited references. The size of the points represents the citation frequency. A line between two points means that both were cited in one paper. A shorter line indicates a closer link between two papers. Points in the same color belong to the same research direction.) (b) Mapping of cocited journals related to the field. (The 825 points with different colors represent the 825 identified journals. The size of the points represents the citation frequency. A line between two points means that both were cited in one journal. A shorter line indicates a closer link between two journals. Points in the same color belong to the same research direction).

Cocitation analysis indicates the relatedness of items based on the number of times they are cited together. There were 498 references (the minimum number of citations of a reference was over 20 times) which were analyzed by using the VOSviewer (Figure 7(a)). The top five papers with large total link strengths were as follows: Wang et al. [28] (TLS = 2,580), Ogden et al. [29] (TLS = 2,219), Gerdesmeyer et al. [30] (TLS = 2,083), Rompe et al. [31] (TLS = 1,822), and Loew et al. (TLS = 1,757) [32].

#### 3.6.2. Journals

A total of 825 journals of cocitation analysis were analyzed using the VOSviewer (the minimum number of citations from a source was over 20 times). The top five journals with large total link strengths were as follows: Journal of Urology (TLS = 257,587), American Journal of Sports Medicine (TLS = 113,001), Clinical Orthopaedics and Related Research (TLS = 105,960), The New England Journal of Medicine (TLS = 98,494), and Urology (TLS = 96,146) (Figure 7(b)).

### 3.7. Co-Occurrence Analysis

**Figure 8:**
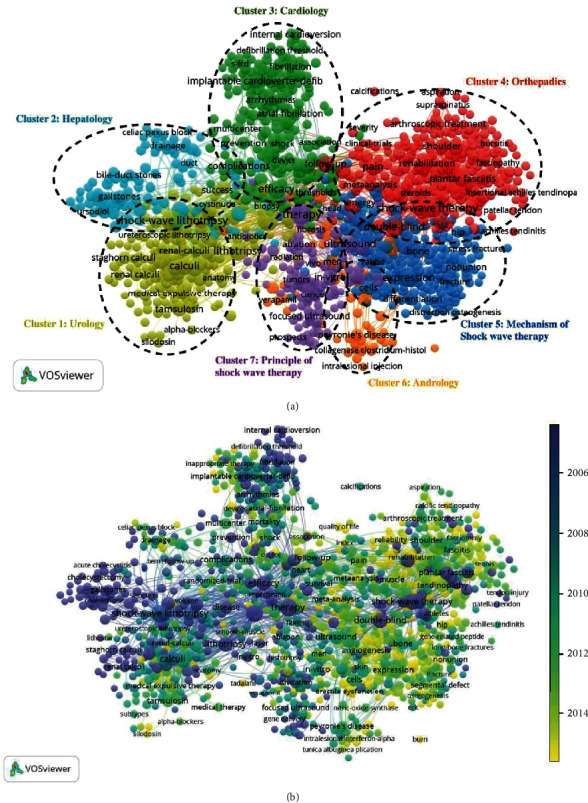
Co-occurrence analysis of global research about shock wave therapy. (a) Mapping of keywords in the research on shock wave therapy—the size of the points represents the frequency, and the keywords are divided into 7 clusters: “urology,” “hepatology,” “cardiology,” “orthopedics,” “mechanism research of shock wave therapy,” “andrology,” and “principle of shock wave therapy.” (b) Distribution of keywords according to the mean frequency of appearance—keywords in purple appeared earlier than those in green and yellow-colored keywords appeared later.

Co-occurrence analysis is a method to build the relationship of items based on the number of publications in which they occur together. The purpose of it is to determine research areas and popular issues, and it is important for monitoring scientific development [22]. The keywords were analyzed by the VOSviewer (the minimum number of occurrences of a keyword was over five). It is shown in Figure 8(a) that the 1,144 included keywords were grouped into approximately 7 clusters: “urology,” “hepatology,” “cardiology,” “orthopedics,” “mechanism research of shock wave therapy,” “andrology,” and “principle of shock wave therapy.” In the “urology” cluster, the main keywords are shock-wave lithotripsy, management, lithotripsy, calculi, and urolithiasis. In the “hepatology” cluster, the main keywords are stones, disease, extracorporeal shock wave lithotripsy, fragmentation, and chronic pancreatitis. In the “cardiology” cluster, the main keywords are efficacy, trial, complications, surgery, and implantable cardioverter-defibrillator. In the “orthopedics” cluster, the main keywords are shock-wave therapy, double-blind, extracorporeal shock wave therapy, pain, and randomized controlled trial. In the “mechanism research of shock wave therapy” cluster, the main keywords are therapy, ultrasound, in-vitro, shock wave, and in-vivo. In the “andrology” cluster, the main keywords are erectile dysfunction, prevalence, Peyronie's disease, natural-history, and placebo. In the “principle of shock wave therapy” cluster, the main keywords are expression, cells, model, angiogenesis, and ischemia. These results demonstrated the most core themes of the shock wave therapy research field up till now.

Keywords were color-coded by VOSviewer based on the average time they appeared in all included publications. The blue color means the keyword appeared early, and yellow-colored keywords appeared later.  Figure 8(b) shows that most of the studies focused on “urology,” “hepatology,” “cardiology,” and “principle of shock wave study” before 2014. However, the recent development trends show that the clusters of “orthopedics,” “andrology,” and “mechanical research of shock wave therapy” will be extensively concerned in the future.

## 4. Discussion

### 4.1. Global Trends in Shock Wave Therapy

Bibliometrics and visualized analysis can not only describe the current research status but also monitor trends in the field [33]. Bibliometrics and visualized analysis can not only describe the current research status but also predict future research directions in the field. Therefore, our study was intended to investigate the shock wave therapy concerning global trends of publications and the contribution of countries, institutions, and research focus through this way. In recent years, the advancement in the field has been a constantly developing area of research. The number of publications is increasing every year. As shown in this study, a total of 79 countries were shown to have published articles in this field. Furthermore, the co-occurrence analysis could indicate the possible research orientation for the future. Thus, in terms of current results, we predict that more studies on shock wave therapy might be published in the next few years.

### 4.2. Quality and Status of Global Publications

Although the *h*-index has some limits, the *h*-index and the total number of citations represent the academic impact and quality of a nation's publication generally [34, 35]. From our results, the USA made the largest contributions to global shock wave therapy research in terms of a total number of publications as well as total citation frequency and *h*-index. Therefore, the USA could be regarded as a leader in this field. It is worth noting that China ranked fourth in the total number of publications, but both its *h*-index and total citation frequency ranked only twelfth. This difference could be explained by the fact that the Chinese academic evaluation system has been focusing more on the quantity rather than the quality of publications. With the gradual increase of scientific research funds, the National Nature Science Foundation of China has ranked the third in the world, which shows a greater possibility for China's publication quality improving in the future.

The Journal of Urology, Ultrasound in Medicine and Biology, Urology, and Journal of Endourology published the most studies on shock wave therapy, which is easy to explain: shock waves were first used in the clinical practice as a method of urethral lithotripsy. However, except urology, the rest of the top 20 journals were almost all about cardiology and orthopedics. That means the applied range of shock waves has been extended. The journals in the list (Figure 4(a)) may be the main publishing channels for future publications in this field.

Almost all of the top 20 institutions were from the top five countries, which means that the first-class research institutions have been playing an important role in the development and ranking of the national academic level. At the same time, Figure 4(c) listed the 20 authors who have published the most articles about shock wave therapy, and they are considered to be pioneers in this domain. In the future, their research could have an important impact on the development of shock wave therapy, which deserves more concern as well.

In this study, we used bibliographic coupling analysis to establish a similarity relationship between papers with regard to country, institution, and journal. Through the analysis of references, the bibliographic coupling will occur when two articles contain references from the same article or journal. And the result indicated that Ultrasound in Medicine and Biology was the most related journal, and the USA was the leading country in this field. Coauthorship analysis was used to assess cooperation among countries, institutions, and authors. The country/institution/author would be more likely to cooperate with others with the highest total link strengths. The purpose of cocitation analysis was to investigate the impact of research by calculating the number of citations. Shock wave therapy induces neovascularization, which, as a milestone study, had the greatest total frequency of cocitation. The Journal of Urology was the journal with the highest citation frequency in the shock wave therapy field.

### 4.3. Research Focus on Shock Wave Therapy

We used co-occurrence analysis to identify future trends and hotspots. The map of the co-occurrence network was created based on the keywords of all titles and abstracts of the included studies. As shown in Figure 8(a), seven research trends were noticed, including urology, hepatology, cardiology, orthopedics, mechanism research of shock wave therapy, andrology, and the principle of shock wave therapy. Although this result was consistent with common sense in this field, it could make the orientation of future research. Additionally, it is very important to comprehend the development of shock wave therapy in different subjects. The intersection and conformity among subjects are the growing direction of medicine. Therefore, investment and high-quality research in these seven fields are still needed in the future. The overlay visualization map was the same as the co-occurrence map, but the color of the project is different. The color bar indicates how the scores were drawn to the color. It is an important method to predict the research direction and has great significance. From the overlay visualization map (Figure 8(b), colors indicate publication years), orthopedics, andrology, and mechanism research of shock wave therapy (yellow color) may be the next hot topics in this field. Consistently, the mechanism study lagging behind the clinical effect has attracted more scholars to do basic research. Recently, Matsuda et al. pointed out that shock wave therapy promoted BDNF expression and improved functional recovery after spinal cord injury in rats [36]. The promising result provided a theoretical basis for the application of shock waves in “sensitive” organs (nerve and brain). And it will attract more attention to this field.

Based on this study, the increasing number of publications indicated that shock wave therapy, as a noninvasive method, has a crucial position in medicine. Through the bibliometric and visualized analyses, researchers and investment departments commendably understood the current research status in the field, which provides a reference for their future research direction.

### 4.4. Strengths and Limitations

Our study first evaluated the status and trends of the studies about shock wave therapy via bibliometric and visualized analyses. It provided a reference for understanding the development of shock wave therapy and compared the contributions of different scholars, journals, research directions, and countries. Meanwhile, our results help researchers to know the results of interdisciplinary researches about shock wave therapy. But some limitations have to be mentioned. Firstly, the low number of publications in languages other than English limits the collection of scientific data, leading to language bias. Otherwise, differences may exist between the real world and the present results. For example, other databases (PubMed, Ovid, and Google Scholar) were not analyzed, which could increase the number of articles, authors, and journals.

## 5. Conclusion

This study showed the current situations and global trends in shock wave therapy. The United States is the leading country in both the total number of publications and the total citation frequency. The Journal of Urology published the most papers related to this issue. More studies on shock wave therapy will be published in the next few years. In particular, studies about orthopedics, andrology, and mechanical research of shock wave therapy will be the next popular hotspots and attract more attention in the future.

## Data Availability

The data used to support the findings of this study are included in the article.
